# Deep Learning for Automated Analysis of Cellular and Extracellular Components of the Foreign Body Response in Multiphoton Microscopy Images

**DOI:** 10.3389/fbioe.2021.797555

**Published:** 2022-01-25

**Authors:** Mattia Sarti, Maria Parlani, Luis Diaz-Gomez, Antonios G. Mikos, Pietro Cerveri, Stefano Casarin, Eleonora Dondossola

**Affiliations:** ^1^ Department of Electronics, Information and Bioengineering, Politecnico di Milano University, Milan, Italy; ^2^ David H. Koch Center for Applied Research of Genitourinary Cancers and Genitourinary Medical Oncology Department, The University of Texas MD Anderson Cancer Center, Houston, TX, United States; ^3^ Department of Cell Biology, Radboud University Medical Center, Nijmegen, Netherlands; ^4^ Rice University, Dept. of Bioengineering, Houston, TX, United States; ^5^ Center for Computational Surgery, Houston Methodist Research Institute, Houston, TX, United States; ^6^ Department of Surgery, Houston Methodist Hospital, Houston, TX, United States; ^7^ Houston Methodist Academic Institute, Houston, TX, United States

**Keywords:** foreign body response, deep learning, U-Net, intravital multiphoton microscopy, image analysis

## Abstract

The Foreign body response (FBR) is a major unresolved challenge that compromises medical implant integration and function by inflammation and fibrotic encapsulation. Mice implanted with polymeric scaffolds coupled to intravital non-linear multiphoton microscopy acquisition enable multiparametric, longitudinal investigation of the FBR evolution and interference strategies. However, follow-up analyses based on visual localization and manual segmentation are extremely time-consuming, subject to human error, and do not allow for automated parameter extraction. We developed an integrated computational pipeline based on an innovative and versatile variant of the U-Net neural network to segment and quantify cellular and extracellular structures of interest, which is maintained across different objectives without impairing accuracy. This software for automatically detecting the elements of the FBR shows promise to unravel the complexity of this pathophysiological process.

## 1 Introduction

The penetration of a foreign material inside a host organism activates a cascade of events, defined as foreign body response (FBR), aimed to minimize its negative impact (Sheikh et al., 2015; Veiseh and Vegas, 2019). This stepwise process initiates with vascular damage and absorption of plasma proteins to the object, followed by an acute inflammation led by neutrophils and a chronic phase sustained by macrophages and foreign body giant cells (Anderson et al., 2008; Dondossola et al., 2016; Witherel et al., 2018; Veiseh and Vegas, 2019; Gurevich et al., 2020; Dondossola and Friedl, 2022). In parallel, resident stromal cells, such as fibroblasts, are recruited and activated, leading to the formation of a fibrotic capsule that shields the host from the material (Veiseh and Vegas, 2019). Designed by nature to protect healthy tissues from foreign assault, the FBR has more recently emerged as a clinical problem for the functionality of implanted medical devices because inflammation and fibrosis can cause their degradation and create a physical barrier that compromises performance. As a result, sensors, pacemakers, prostheses, and scaffolds used in tissue engineering and regenerative medicine can experience malfunction and failure (Prakasam et al., 2017). Given the impact of the FBR on implanted medical devices, several strategies have been proposed to reduce the resulting inflammatory and fibrotic response, including modification of material-intrinsic properties (e.g., size, shape, texture, and functionalization) (Zhang et al., 2013; Veiseh et al., 2015; Vegas et al., 2016; Shayan et al., 2018; Zhang et al., 2021) and pharmacological interference (Galeska et al., 2005; Klueh et al., 2005; Morais et al., 2010; Kastellorizios et al., 2015; Moore et al., 2016; Morris et al., 2017). Despite improving outcome, none of these approaches have resulted in complete and long-lasting control of FBR (Veiseh and Vegas, 2019). In order to better understand the mechanisms underlying the FBR and further identify strategies that effectively reduce this phenomenon, relevant preclinical models need to be developed and outcome properly monitored, analyzed, and quantified.

**GRAPHICAL ABSTRACT:**
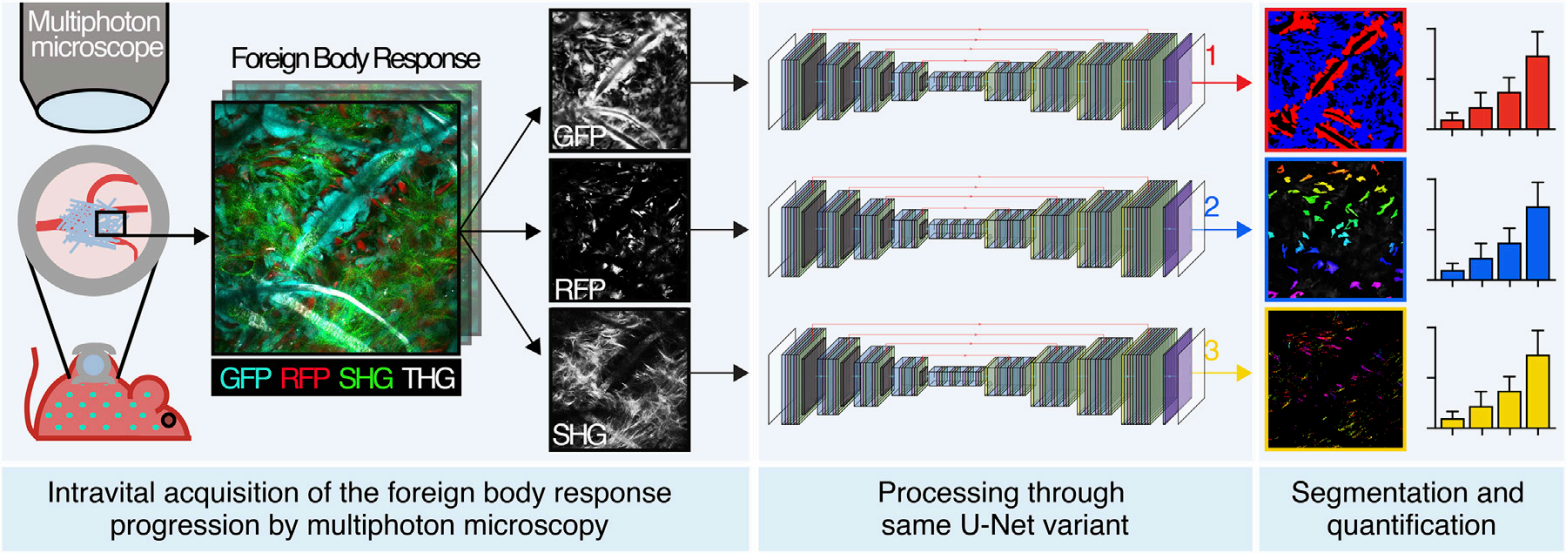


Experimental approaches probing the FBR in small animals are mostly based on *ex vivo* endpoint analysis (e.g., immunohistochemistry). Although informative, this strategy lacks sensitivity and time resolution to characterize dynamic disease progression. Intravital multiphoton microscopy recently emerged as a powerful tool to complement *ex vivo* analyses by providing mechanistic, three-dimensional (3D), and time-resolved multiparametric insights about implant integration and failure (Dondossola and Friedl, 2021). This application generates high-content biological images, which are mostly analyzed manually, including individual cell counting by eye inspection, manual measurement of distances, and qualitative identification of cellular subtypes, which imply several disadvantages. Such manual procedures, indeed, are extremely time-consuming (up to several days or weeks for specific analysis), display low accuracy when performed serially for a huge number of images, and are operator-dependent (non-univocal), leading to a lack of standardization. For these reasons, the need for an automated approach is evident. Since the missing step in the workflow implies to emulate choices of human experts, who had learned how to distinguish structures by seeing examples and not following detailed low-level rules, deep learning was identified as a solution to perform semantic segmentation.

Deep learning has been widely adopted for semantic segmentation in the biomedical domain. U-Net is a fully convolutional model used in microscopy imaging (Ronneberger et al., 2015), which requires a relatively limited amount of training samples and allows reconstructing high-resolution segmentation masks from low-resolution encoded representations. This architecture experienced widespread adoption for biomedical semantic segmentation tasks, usually with small deviation from the original design (Al-Kofahi et al., 2018; He et al., 2021; Kose et al., 2021). As an example, *ex vivo* cellular nucleus segmentation has been performed by combining three U-Net–like branches with custom layer blocks (Zhao et al., 2020). Similarly, dense layer blocks and dense concatenation were employed to increase the architecture depth and combine features for fine detail reconstruction and localization in *in vivo* multiphoton microscopy images (Cai et al., 2020).

In this study, we develop an integrated computational pipeline for automated segmentation and analysis of the cellular and extracellular components of the FBR based on an innovative and versatile variant of the U-Net neural network. Our work implements automated tools that allow members of the research community (e.g., biologists, materials scientists, biomedical engineers, and implant pathologists) to investigate and quantify the progression of the FBR more efficiently than manual analysis. We also demonstrate how versatile a variant of the base U-Net architecture is across different objectives, with no hyperparameter tuning and in data-critical (30–50 samples) microscopy image segmentation tasks, addressing recent concerns in the literature. Finally, we define novel convolutional kernels and multiclass Dice loss.

## 2 Materials and Methods

### 2.1 Mouse Model Generation

Animal studies were approved by the Institutional Animal Care and Use Committee of the University of Texas, MD Anderson Cancer Center, which is accredited by the Association for Assessment and Accreditation of Laboratory Animal Care. Mice older than 8 weeks male or female were housed with a maximum of five animals per cage in a state-of-the-art, air-conditioned, and specific pathogen–free animal facility and all procedures were performed in accordance with the NIH Policy on Humane Care and Use of Laboratory Animals. C57BL/6-Tg (UBC-GFP) 30Scha/J mice, which ubiquitously express green fluorescent protein (GFP), were from Jackson Lab. C57BL/6-Tg(Acta2-DsRed)1Rkl/J mice, which express DsRed red fluorescent protein (RFP) under the alpha smooth muscle actin, αSMA, promoter, were a gift from Dr. Raghu Kalluri, The University of Texas MD Anderson Cancer Center (LeBleu et al., 2013). To establish a mouse model that displays GFP immune cells and RFP-activated myofibroblasts, bone marrow transplantation was performed, as previously described (Dondossola et al., 2013). Briefly, lethally irradiated (5.5 Gy, twice, with 3 h of recovery time in between) αSMA-RFP mice were infused with bone marrow cells derived from GFP donors (the content of 1 tibia + 1 femur/lethally irradiated mouse), generating an αSMA-RFP/GFP model. Bone marrow engraftment was monitored 1 month posttransplant by *ex vivo* MPM analysis. Mouse tibiae were fixed in PFA 4% for 1 day, decalcified in EDTA 0.5 M, pH 7.5, for 4 days, and sliced (300 µm thick) using a vibratome. Circulating levels of white and red blood cells, platelets, hematocrit, and hemoglobin in both bone-transplanted and non-transplanted control mice were analyzed. Prior to blood collection, the mice were anesthetized using 3–4% isoflurane. When the mice were completely unconscious, a heparinized capillary tube was inserted into the medial canthus of the eye to puncture the tissue and enter in the sinus. Once the required volume of blood was collected (∼100 μL in a tube with 10 μL EDTA), the capillary tube was removed, and bleeding was stopped by applying gentle pressure with a gauze sponge. The blood collected was then diluted 1:10 in PBS and analyzed for leukocyte counts and blood parameters on an ABX Micros 60 hematology analyzer.

### 2.2 Material Generation and Implantation

To fabricate scaffolds, polycaprolactone (PCL; 43 kDa, Polysciences; Warrington, PA) was melted at 85°C and printed at a collector velocity of 40 mm s−1, 5.0 kV, 1.0 bar, at a distance of 10 mm using a 3DDiscovery Evolution printer, RegenHU, Switzerland, located in a laminar flow hood. Scaffolds, designed using computer-aided design software BioCAD (Regenhu, Switzerland), had a filament width of 35 μm and 90% porosity. They were stored in 70% ethanol until their application.

**FIGURE1:**
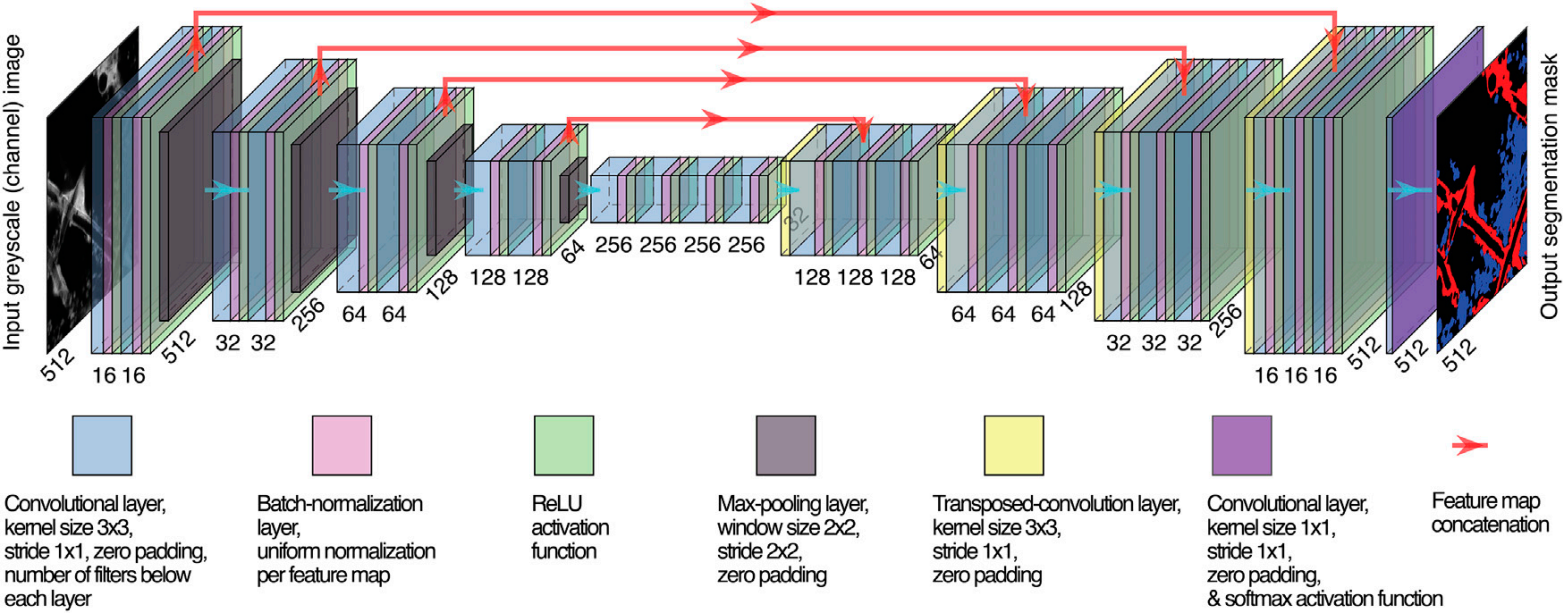
U-Net variant, network architecture. The same number of layer blocks is employed for the encoder and the decoder (in the example, 4) and the number of filters in each convolutional layer, but the last one maintains the same ratio with respect to the one of the first layer (16, in the example). Spatial dimensions of feature maps and numbers of filters are reported, respectively, diagonally and horizontally.

The scaffolds were implanted in parallel to the deep dermis/subcutis interface in mice within a dorsal skinfold chamber system, an optical imaging window that allows for *in vivo* inspection in real time (Figure 1B), as previously described (Dondossola et al., 2016). Longitudinal monitoring of the scaffolds started 4 days postimplantation and proceeded up to day 14.

For clodronate treatment experiments, mice implanted with the scaffold in the dorsal skinfold chamber received clodronate liposomes (1 mg/mouse, 200 μL, intravenously, following the manufacturers’ instructions, Liposoma) every two to three days, starting three days before scaffold implantation to deplete macrophages by the day of implantation, as reported (Dondossola et al., 2016).

### 2.3 Image Acquisition, Manual Analysis, and Comparison of Outcome to Other Image Analysis Platforms

Non-linear multiphoton microscopy was used to dissect the three-dimensional spatial organization and fate of scaffold integration. A custom-made multiphoton microscope with three different titanium–sapphire lasers and two optical parametric oscillators (yielding a tunable range of excitation wavelengths between 800 and 1300 nm) was employed. Multispectral detection of 3D stacks was performed using up to five photomultipliers and three excitation wavelengths in consecutive scans, to separate the following excitation and emission channels: GFP (920 nm; 525/50 nm), RFP (1090 nm; 595/40 nm), second harmonic generation (SHG; 1090 nm; 525/50 nm), and third harmonic generation (THG; 1180 nm; 387/15 nm) (Dondossola et al., 2016).

The volumes acquired were characterized by the same constant in-plane physical spatial resolutions of 360 × 360 μm, 1064 × 1064 px, while the depth physical resolution, in between slices, was 5 μm, for a maximum depth of 250–300 µm.

Scaffold-associated and interstitial cells were manually segmented as follows:

1) Scaffold-associated cells: This subset consists of a discrete layer of cells characterized by close contact with the scaffold (which extends as a unique body up to 20–40 µm distant from the fiber), showing a relatively higher brightness;
2) Interstitial cells: These are the immune cells that do not present any direct association with the scaffold fiber. We did not distinguish specific immune infiltrating cells (e.g., monocytes, macrophages, lymphocytes, and granulocytes).

Each cell subset was outlined, and the area occupied was calculated by ImageJ (Schindelin et al., 2012; Schneider et al., 2012). The number of fibroblasts was manually counted.

Comparison of the outcome to that of other image analysis platforms was performed as follows: 5 images with three different cell density settings were analyzed by U-Net, Icy (de Chaumont et al., 2012) and arivis Vision4D 3.5 (arivis AG) and compared to a manually defined ground truth. For fibroblasts, the analysis was performed as follows: 1) Icy: HK-Means, five intensity classes, min. object size 2000 px, max. object size 6000 for low–cell density images; min. object size 200px, max. object size 100000 for medium– and high–cell density images. 2) arivis Vision4D 3.5: the machine learning algorithm was trained with examples of fibroblasts (Class 1, 200 cells) and background (200 examples). For immune-infiltrating cells, the analysis was performed as follows: 1) Icy: HK-Means, five intensity classes, min. object size 100px, max. object size 30,000. 2) arivis Vision4D 3.5: this machine learning algorithm was trained with examples of scaffold-associated cells (Class 1, 45 images), interstitial cells (Class 2, 45 images), and background (45 images). The total area occupied by scaffold-associated and the interstitial cells was determined, and the percentage of area occupied by each population was then calculated.

### 2.4 U-Net Variant Customization

We employed a customized U-Net variant (Ronneberger et al., 2015), the structure of which is shown in Figure 1. Batch normalization (Ioffe and Szegedy, 2015) was carried out between any convolutional layer and its subsequent ReLU activation function. Learnable transposed convolution layers (Dumoulin and Visin, 2016) were employed as upsampling layers. We used the same variant across all tasks, adopting 16 filters in the first convolutional layer and five encoding/decoding layer blocks. The neural network was trained for 300 epochs with 8-sized mini-batches to guarantee training stability, generalization of performance, and a contained RAM requirement for training (Masters and Luschi, 2018) yet allowing for a meaningful sample size for batch normalization.

### 2.5 Segmentation of Immune Infiltrating Cells

Our U-Net variant was trained to accomplish multiclass segmentation to differentiate within the input images the scaffold-associated cells and interstitial cells (as described in Section 2.3) and the background (the acellular content, including both scaffold framework and the non-visible substrate; Supplementary Figure S1).

To train the network, we selected 36 non-consecutive GFP images from our 3D images dataset ensuring sample variability and independence. We resized the images from 1064 × 1064 to 512 × 512 pixels resolution *via* bicubic interpolation to reduce the computational burden and to capture large enough spatial patterns in the last encoding layer, allowing for more global structure observation. Since training images were acquired from different volumes, they presented different intensity ranges and exposures. To cope with this issue, we normalized the original images by linearly remapping the intensity values in the [0; 1] range to obtain the same distribution for each one. Images were labeled to generate the target masks using Amira software under the supervision of an expert biologist. Images and labels were randomly split between training (25 samples) and validation sets (11 samples). We used data augmentation on both datasets to create a transformed version of source images to increase the number of samples and labels by a factor of 8. The augmentation was performed after training *vs.* validation splitting to ensure the independence of the two datasets. We used a combination of mirroring and rotation with π/2 step in the [0; 2π] range to increase the training dataset from 25 to 200 and the validation dataset from 11 to 88. We did not employ any elastic deformation or resizing to augment images nor to invalidate shape and scale of the structures, respectively, as both represent relevant factors that the algorithm should not become invariant to. We did not add arbitrary noise distributions to image pixels when augmenting images as a low signal-to-noise ratio had never been encountered during acquisitions and was unexpected at inference time. Accordingly, we assumed any kind of unexpected image artifact to be possibly informative and not distinguishable from acquisition noise.

The neural network was trained as described to minimize an objective on the training set, finally evaluating IoU metrics on the validation set.

We defined the loss function as the sum of a multiclass extension of the standard Dice Loss (L_M-DICE_) and a regularization term to reach a trade-off between minimizing L_M-DICE_, which reflects the adherence to segmentation labels and constraining the weights that prevents overfitting. The loss function is given as follows:

$$L=L_{M-DICE}+\frac{\lambda }{2}\|\omega {\|}_{2}^{2},$$

where λ=0.01 gives the regularization term a 1% relative importance with respect to correctly accomplishing a satisfying segmentation of the training dataset.

Similar to published work (Novikov et al., 2018; Shen, 2018; Abraham and Khan, 2019), (L_M-DICE_) can be written as follows:

$$L_{M-DICE}=\frac{1}{N_{S}}\underset{S}{\sum }\left(1-\overset{\;}{\underset{C}{\sum }}\alpha_{C}\left(\frac{2\sum_{x\in S}^{\;}y_{C}\left(x\right){\hat{y}}_{C}\left(x\right)}{\sum_{x\in S}^{\;}y_{C}\left(x\right)+\sum_{x\in S}^{\;}{\hat{y}}_{C}\left(x\right)}\right)\right).$$

The standard Dice Loss was computed for each class (C) by considering all remaining classes as background and evaluating the associated pixel-wise predicted ( $\hat{y_{c}}$ ) and expected ( $y_{c}$ ) probabilities. The resulting term was weighted by a dedicated class balance coefficient ( $\alpha_{c}$ ) to avoid classes richer in pixels to be favored in the overall loss. These coefficients were computed by measuring the occurrences of all pixels of respective classes in the training set and normalized to sum to 1. The loss was finally generalized to a multiclass case by subtracting 1 the sum of weighted terms of each class. The resulting value for each image was averaged over all $N_{s}$ mini-batch samples (S).

The presented loss function was minimized by Adam optimization algorithm (Kingma and Ba, 2014) by employing a sigmoid-like base learning rate (l_r_(E)), defined as a function of the epochs (E) to bound the extreme values. l_r_(E) ranged from an initial value lr0 = 0.001 at the beginning of training (first epoch, E_0_ = 0) to a final value l_r∞_ = 0.0001 reached toward the end of training (E_∞_ = 200) in correspondence of which saturation was reached. Accordingly, the trend writes as follows:

$$l_{r}\left(E\right)=\left(l_{r0}-l_{r\infty }\right)*\frac{1}{1+e^{s\left(E\right)}}+l_{r\infty }$$

with

$$s\left(E\right)=\frac{E-\left(\frac{E_{\infty }-E_{0}}{2}\right)}{\frac{E_{\infty }-E_{0}}{10}}$$

and it is shown in Supplementary Figure S2.

Once the network was trained, any arbitrary image in our dataset could be normalized and resized as before to feed it and obtain the predicted masks. After nearest neighbor mask upsampling, we measured the area of each class of interest to quantify the cellular species content within the image. The network was implemented in Python through TensorFlow library (https://www.tensorflow.org).

### 2.6 Fibroblast Segmentation and Quantification of Parameters

To train the network, we selected 36 RFP fibroblast-rich images from our 3D dataset. We performed a 3-step image preprocessing including 1) normalization, 2) gamma-correction, and 3) histogram-stretching.

Normalization was performed as in task 1. Gamma-correction remapped the intensities by expanding the lower values and compressing the higher ones to highlight the underexposed structures by increasing their contrast. The gamma value was empirically set to 0.5. Histogram stretching was independently performed for each image to make its intensity distributed over the entire normalized range. The lowest and the highest levels associated with histogram counts above a 50-pixel threshold were, respectively, remapped to 0 and 1, and the remaining levels followed the linear stretching imposed by these extremes. Additionally, levels mapped outside the [0; 1] range were saturated to the extremities.

Labels were manually created and, along with respective samples, they were resized, augmented, and split between training (264 images) and validation (120) as done in task 1.

Based on Ronneberger et al. (2015), an optimal separation of close or adjacent fibroblasts was achieved by using a loss function devoted to emphasize cell separation. Each label was associated with a map assigning a weight to each pixel based on its importance to achieve a correct segmentation of adjacent cells. Pixels belonging to fibroblasts were set to have zero weight, while background pixels were assigned a value depending on their distance from the two nearest cells, where cells were extracted as 8 connected clusters from labels. In this way, the lower the distance, the higher the weight, the more relevant the pixel is. The value was computed as a Gaussian of the sum of the two distances of interest to constrain weight magnitudes. Accordingly, each pixel position ( x ) was assigned a boundary weight ( $w_{B}\left(x\right)$ ) that is written as follows:

$$w_{B}\left(x\right)=\{\begin{array}{c} 0,\;if\;x\notin background\\ \;ae^{-\frac{{\left(d_{1}\left(x\right)+d_{2}\left(x\right)\right)}^{2}}{2\sigma^{2}}},\;if\;x\in background \end{array},$$

where $d_{1}\left(x\right)$ and $d_{2}\left(x\right)$ are the Euclidean distances between x and the two nearest cells; the amplitude ( a ) of the Gaussian was empirically set to 100, while the standard deviation ( σ ) was set to five pixels according to Ronneberger et al. (2015).

To tackle class imbalance, the weight map presented with (4) was corrected by adding a different class importance coefficient depending on the category each pixel belonged to. A background importance ( $i_{B}$ ) and a foreground importance ( $i_{F}$ ) were defined as the mean value of ( $w_{B}\left(x\right)+1$ ) associated with background and foreground pixels, respectively, over the entire training set. Thus, $i_{B}=1$ , yielding unitary final weights for all foreground pixels, that is, a neutral weighting. The resulting class boundary weight map ( $w_{C-B}\left(x\right)$ ) is written as follows:

$$w_{C-B}=\{\begin{array}{c} w_{B}\left(x\right)+i_{F}=1,\;if\;x\notin background\\ w_{B}\left(x\right)+i_{B},\;if\;x\in background \end{array}.$$

A pixel-weighted version of the traditional binary cross-entropy (BCE) was chosen as loss function to allow all pixels to be weighted by their respective class boundary relevance, as follows:

$$L_{w-BCE}=\frac{1}{N_{S}}\sum_{s}\frac{\sum_{x\in S}w_{C-B}\left(x\right)L_{BCE}\left(x\right)}{\sum_{x\in S}w_{C-B}\left(x\right)},$$

where L_BCE_ is the traditional BCE loss multiplied by the respective weight ( $w_{C-B}$ ) and averaged on all pixels (x) of all N_s_ samples (S) in the mini-batch.

Since the maximum amplitude of weights was arbitrarily set, discretionary importance was attributed to segmenting borders of adjacent fibroblasts, rather than their overall structure. As IoU metric represents only the latter, a custom metric was introduced as a weighted average of pixel-wise probability likelihoods based on the same class boundary weights:

$$M=\frac{1}{N_{S}}\underset{S}{\sum }\frac{\sum_{x\in S}w_{C-B}\left(x\right)\left(y\hat{y}+\left(1-y\right)\left(1-\hat{y}\right)\right)}{\sum_{x\in S}w_{C-B}\left(x\right)}.$$

Symbolism is maintained as in (6), with the metric reflecting an inverted version of the employed loss bounded to the [0;1] range.

Training was performed by minimizing the L2-regularized L_BCE_ by Adam optimizer as described for task 1. The network was employed for inference upon satisfying metric evaluation on the validation set.

After output mask upsampling *via* nearest neighbor, fibroblasts were separated by connecting together foreground pixels assigned to the same entity. Two pixels were classified as linked if they were 8-connected, that is, they exhibited adjacent edges or corners. Finally, clusters of pixels with an area lower than 200 were discarded. The number of fibroblasts, their mask, area, centroid coordinates, and inter-fibroblast distance were extracted.

### 2.7 Quantification of Collagen Orientation

#### 2.7.1 Scaffold Segmentation

To train the network, 47 images were used that included two channels (SHG and THG, for collagen and scaffold, respectively). The selected samples were resized, preprocessed, manually labeled under expert biologist supervision, split, and augmented via the same rationale used for fibroblast segmentation, obtaining 256 training and 120 validation samples. The standard Dice loss was regularized and minimized relying on the same optimizer as in task 1, and IoU was evaluated on the validation set at the end of training before deployment.

#### 2.7.2 Collagen/Scaffold Orientation Detection

We detected the orientation of the structures of interest with an angular resolution of 1° by extending compass mask technique (Robinson, 1977) to any arbitrary angular resolution. Accordingly, we created 360 custom kernels for edge detection convolutional filtering. Each kernel was defined to yield the highest convolutional output for boundaries oriented as the respective linear edge represented by its coefficients. The chosen kernel size was 33 × 33 pixels, corresponding to an 11.2 × 11.2 μm physical neighbor, coherently with Bancelin et al. (2014). Kernel coefficients were assigned the respective pixel distance between their position and the line through the kernel center with desired orientation (Supplementary Figure S3A). Next, the coefficients were remapped so that values between 0 and 1 were unvaried; values between 1 and 2 were linearly remapped between 1 and 0, respectively; and values above 2 were set to 0 (Supplementary Figure S3B). The coefficients on the clockwise side of the respective central line were multiplied by -1 (Supplementary Figure S3C). Only the coefficients within a 15 pixel radius were maintained by setting the others to 0 (Supplementary Figure S3D). Finally, to prevent inter-kernel bias and ensure that the coefficients in each kernel sum up to 0, all positive and negative coefficients in each kernel were normalized to sum up, respectively, to 1 and -1 (Supplementary Figure S3E).

The THG scaffold image was resized and preprocessed to feed the trained network and obtain the predicted mask. The latter was upsampled via nearest neighbor interpolation to restore the original size. Next, a single iteration of binary opening with a 20 × 20 pixel elliptical structuring element was applied to remove spurious objects in the mask. Twenty iterations of binary dilation with a 3 × 3 elliptical element were applied to smooth the jagged scaffold boundaries and ensure the mask entirely covers the scaffold boundaries. Finally, the mask was skeletonized following Lee’s algorithm (Zhang and Suen, 1984) and three iterations of binary dilation with a 2 × 2 elliptical element were applied to ensure robustness of the scaffold borders’ directionality.

The resulting mask was convolved with the kernels, yielding 360 feature maps. Each pixel orientation was equal to the one of the kernel with maximum output if the latter was higher than a threshold empirically set to 0.5 or considered as not oriented otherwise.

Pixels on the width-4 borders were considered not oriented to avoid artifacts. Orientations differing 180° were not discerned because they represent opposite intensity gradients across the same edge orientation. Finally, the orientation with the highest number of pixels was chosen to be the preferential one.

To detect collagen orientation, each slice channel was preprocessed analogously to scaffolds, and the same convolutional filtering was applied. Pixel orientations were defined with a 0.06 threshold on the feature map magnitude. Orientations of pixels falling outside the non-skeletonized dilated scaffold mask were discarded to avoid scaffold artifacts, for example, collagen fibril edges. Additionally, pixels with a variance over the top 36 orientation magnitudes lower than 0.35*10^−3^ were discarded to exclude weak orientations that produced similar maximum outputs across several kernels.

Pixel clusters with an 8-connectivity area lower than 15 pixels were considered noisy and discarded. The absolute and scaffold-relative collagen orientations will be presented using dedicated histograms. The histogram trends were filtered *via* a 7-length window moving averaging to smooth noisy spikes without external padding, given the periodicity of angles.

### 2.8 Statistical Analysis

Statistical analysis was performed using GraphPad Prism 7.0 (GraphPad Software, San Diego, CA). Normal distribution was confirmed by using the Shapiro–Wilk test. An unpaired two-sided Student t-test was applied to analyze two populations, while one-way ANOVA, followed by Tukey’s honestly significant difference (HSD) post hoc test, was performed to compare more than two populations. Pearson correlation coefficients were calculated in correlation analyses. All statistical tests were two-sided, and the statistical significance was considered for a *p* value of less than 0.05. Data are shown as mean ± SD.

## 3 Results

### 3.1 Preclinical Modeling of the FBR and Image Acquisition

**FIGURE 2:**
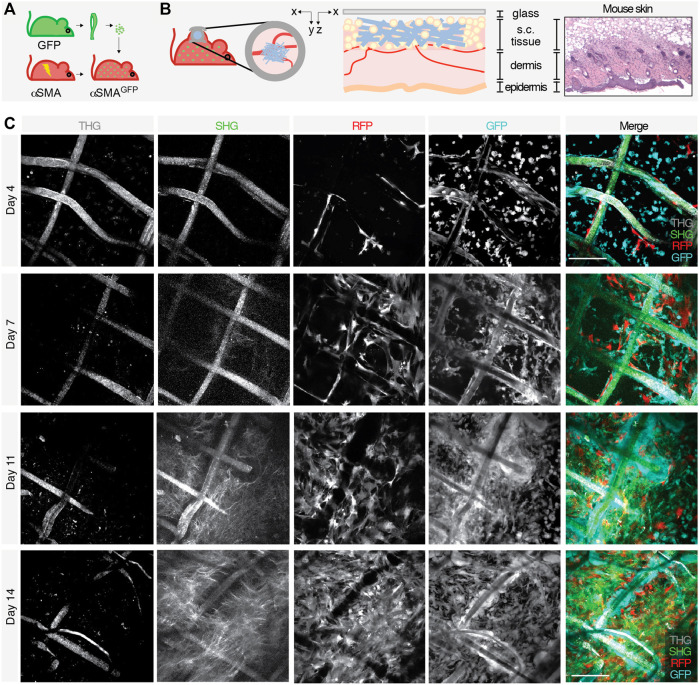
Generation of an *in vivo* model to study the FBR and acquisition of images by longitudinal intravital multiphoton microscopy. Schematic representation of the model showing **(A)** the bone marrow transplant in lethally irradiated αSMA-RFP mice reconstituted with GFP bone-derived cells to generate an αSMA-RFP/GFP mouse and **(B)** scaffold implantation inside the DSFC within the subcutaneous tissue, on top of the dermal layers (xy and xz projections of the implantation site are shown). Right panels show a scheme and the histology (H&E staining) of the mouse skin at the implantation site. **(C)** Longitudinal intravital imaging of the FBR elicited by the PCL scaffold. Single channels and merged pictures at days 4, 7, 11, and 14 post-implantation are shown. THG (gray); GFP-positive cells (cyan); RFP-positive cells (red), and SHG (green). Scale bar, 100 μm.

To monitor the evolution of the FBR over time by intravital microscopy, we established a dual color C57BL/6 mouse model that displays green fluorescent immune cells (GFP) and activated myofibroblasts expressing the red fluorescent protein (RFP) under the promoter of alpha smooth muscle actin (αSMA). To this purpose, the bone marrow of lethally irradiated αSMA-RFP mice was reconstituted with GFP bone-derived cells, generating an αSMA-RFP/GFP mouse (Figure 2A). Engraftment of GFP bone marrow cells was confirmed by *ex vivo* MPM analysis of bones 1 month posttransplantion. Hematological parameters (white and red blood cells, platelets, hematocrit, and hemoglobin) showed no significant differences compared to non-transplanted mice, excluding potential functional issues and confirming full reconstitution (Supplementary Figure S4). The foreign body, consisting of a polycaprolactone (PCL) scaffold, was implanted parallel to the dermis/subcutis interface within a dorsal skinfold chamber system, an optical imaging window implanted on the back of the mouse to allow for *in vivo* inspection in real time (Figure 2B). The progression of the FBR was monitored by 3D multi-position acquisition in the living mouse by non-linear multiphoton microscopy for up to 2 weeks to visualize immune cells (GFP), activated fibroblasts (RFP), scaffold fibers (second and third harmonic generation; SHG and THG), and collagen deposition (SHG). SHG and THG are two label-free, non-linear functions of light captured by the MPM. SHG results from frequency doubling of photons when interacting with non-centrosymmetric molecules and materials, including coiled-coil or polymeric proteins (such as fibrillar collagen) and synthetic polymers (such as PCL). THG originates from frequency tripling of photons when interacting with interfaces that display a mismatch of the refractive index, such as aqueous/lipid rich structures (e.g., lipid droplets and adipocytes) or polymers, including PCL, and water or air (Dondossola et al., 2016; Dondossola and Friedl, 2021). Although autofluorescence emitted from NAD(P) H, flavins, aromatic amino acids, and lysosomes can partially overlap with the emission of GFP, this limited background signal was not detected due to the relatively high levels of GPF expression, which required minimal laser power for excitation and consequent detection. No endogenous tissue autofluorescence that overlap with the detection of other fluorochromes or second and third harmonic generation was identified.

The scaffold became gradually infiltrated by both GFP- and RFP-positive cells, followed by deposition of fibrillar collagen (Figure 2C). In order to segment and quantify these 3D multiparametric images, we developed a versatile U-Net neural variant as extensively described in the Materials and Methods section. Briefly, images of 360 × 360 μm (1064 × 1064 px) acquired every 5 μm for a maximum total depth of 250–300 µm were converted to 512 × 512 px resolution (Figure 1) and cellular and extracellular parameters segmented, as described in the next sections.

### 3.2 Automatic Quantification of Scaffold-Associated and Interstitial Cells

**FIGURE 3:**
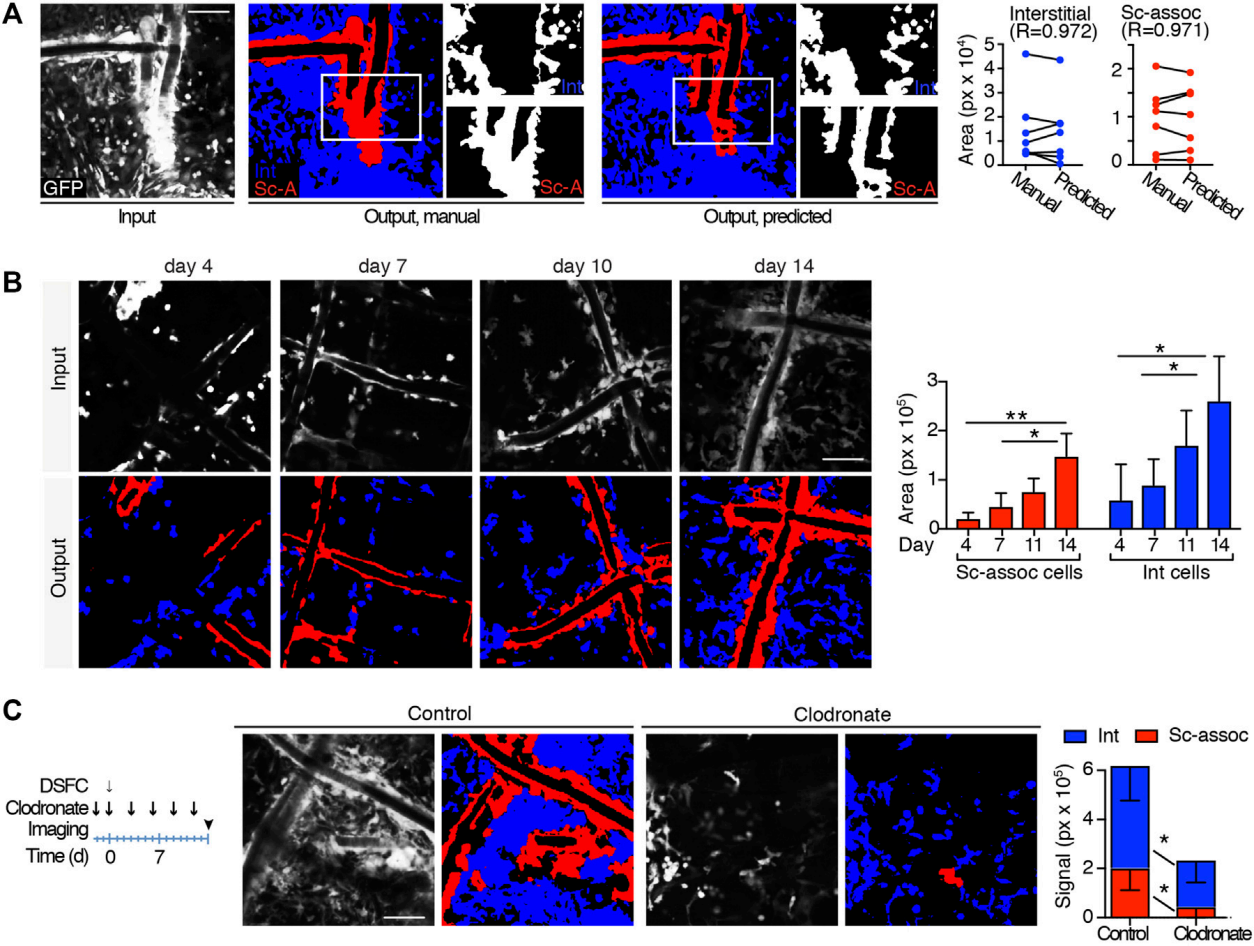
Automatic quantification of scaffold-associated and interstitial cells. **(A)** Segmentation of scaffold-associated cells (red), interstitial cells (blue), and background (black). Left panel, preprocessed input image; central panel, target manual label; right panel, predicted output mask. Insets show single binary masks for interstitial and scaffold-associated (sc-assoc) cells. Pearson correlation between the area of interstitial and scaffold-associated cells in both manual and predicted outputs (obtained from 7 independent images) is shown. **(B)** Automatic quantification of sc-associated and interstitial cells 4, 7, 11, and 14 days post-implantation. Representative images of the GFP-positive cells at the implantation site at all the time points (input), together with their predicted output masks (output) are shown. Histograms quantify the area of sc-associated and interstitial cells at each time point. (*n* = 4 mice, 3 images/mouse). *p < 0.05, **p < 0.01 (unpaired two-tailed Student *t*-test). Scale bar, 100 μm. **(C)** Automatic quantification of sc-associated and interstitial cells in untreated and clodronate liposome–treated mice at day 14. The treatment and imaging schedule is shown. Input representative images and their output masks of FBR in control and clodronate-treated mice are shown. Histograms represent area quantification for sc-associated and interstitial cells in control and treated mice. (*n* = 4 mice/group, 3 images/mouse). *p < 0.05, **p < 0.01 (unpaired two-tailed Student *t*-test). Scale bar, 100 μm.

To automatically quantify the recruitment of immune-infiltrating GFP cells over time, U-Net was trained to perform a multiclass segmentation that differentiates elements in input images. This included 1) scaffold-associated cells—a discrete layer of cells in close contact with the scaffold fiber extending as a unique body up to 20–40 µm of distance, showing a relatively higher brightness; 2) interstitial cells—representative of infiltrating leukocytes which do not present any direct association with the scaffold fiber; and 3) the remaining acellular background. The segmentation network maximized intersection over union (IoU) values [a metric to measure the accuracy of an object detector (Rezatofighi, 2019)] over the validation set to 0.533, 0.580, and 0.902 for scaffold-associated cells, interstitial cells, and background, respectively, showing good prediction ability. To biologically validate the performance of our trained automatic segmentation tool, we compared the area of scaffold-associated and interstitial cells in 7 manually labeled images spanning different cellular densities and correlated the outcome with predictions. The area occupied by both cell subsets in manually labeled images or predicted semantic segmentation strongly correlated (R > 0.9), confirming the accuracy of this analysis (Figure 3A). To further validate our segmentation approach on immune-infiltrating GPF cells at low, medium, or high density spanning days 4–14 post-implantation, we compared the outcome deriving from manual quantification, our trained U-Net, arivis Vision4D 3.5 (arivis AG) and Icy (de Chaumont et al., 2012)—two popular image analysis platforms. Manual segmentation, U-Net, and arivis4D 3.5 achieved similar results, further confirming that the criteria identified to segment interstitial and scaffold-associated cells define two distinct populations, and U-Net can accurately distinguish between them. Icy was not able to distinguish any specific subset; alternatively, when comparing the total area occupied by immune-infiltrating GPF cells, Icy performed similarly to the manual quantification and U-Net for cells at low density but showed poor results at medium and high cellularity (Supplementary Figure S5). Semantic segmentation was then applied to analyze GFP-immune cell recruitment to the scaffold at different time points of FBR formation (*n* = 4 mice, 3 images/mouse), showing progressive and significant increase in both infiltrating interstitial cells and scaffold-associated cells, in line with published data (Dondossola et al., 2016) (Figure 3B). Then, to further evaluate the effectiveness of our U-Net variant, semantic segmentation was applied to quantify the area of scaffold-associated and interstitial cells in scaffolds implanted in mice pharmacologically treated with liposomal encapsulated clodronate (dichloromethylene diphosphonate). This agent depletes all the immune cells derived from the monocytic/macrophage lineage and is expected to reduce the overall number of GFP cells recruited by PCL implantation (Dondossola et al., 2016; Van Rooijen and Sanders, 1994). Clodronate significantly decreased the formation of scaffold-associated cells, as compared to control-treated mice (*n* = 4 mice, 3 image/mouse; Figure 3C), suggesting their monocyte/macrophage-derived origin. Interestingly, the amount of interstitial cells decreased as well, confirming that monocytes/macrophages represent a major population of the interstitial cell compartment (Dondossola et al., 2016).

### 3.3 Automatic Quantification of Fibroblasts

**FIGURE 4:**
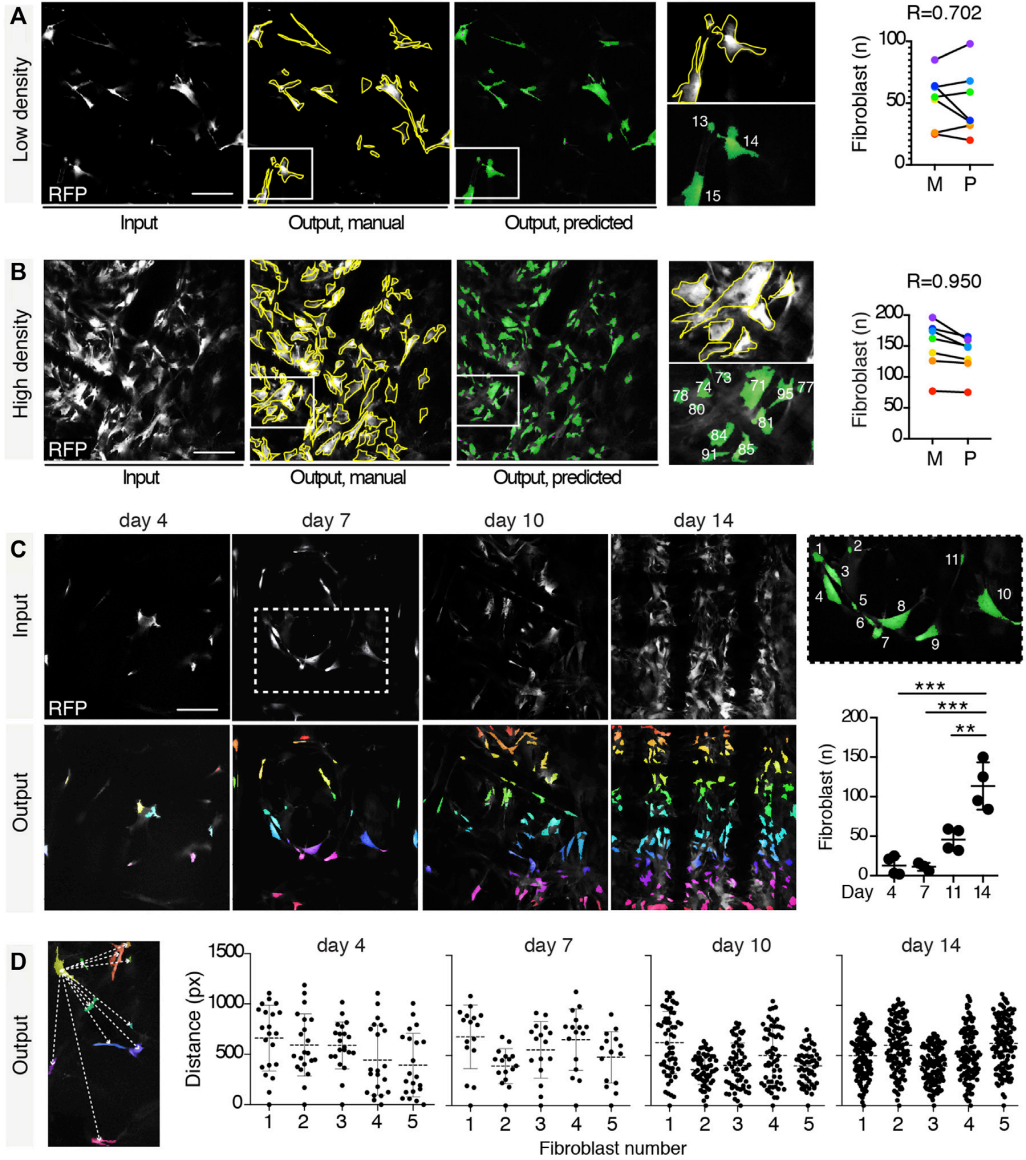
Fibroblast segmentation and extraction of parameters. Segmentation of fibroblasts in a low- **(A)** and a high-density **(B)** setting. Input images in gray, target manual labels with yellow outlines, and predicted segmentation masks in green. Magnifications of fibroblasts manually segmented, and their relative predicted output are shown (each different fibroblast is numbered in white). Pearson correlation between the number of fibroblasts in low- and high-density settings according to the manual and the predicted outputs (obtained from 7 independent images) is shown. **(C)** Automatic quantification of the number of fibroblasts at days 4, 7, 11, and 14 post-implantation. Representative input images for each time point (gray) with relative predicted outputs (rainbow color map). Right panel shows a magnification (inset, day 7) of the predicted output (each fibroblast is numbered in white). Right graph shows the quantification of the number of fibroblasts at each time point obtained by automatic count (*n* = 4 mice, three images/mouse). *p < 0.05, **p < 0.01, ***p < 0.001 (unpaired two-tailed Student’s *t*-test). Scale bar, 100 μm. **(D)** Extraction of mutual distances between centroids. Left panel shows a representation of the distance (white dashed lines) between the centroid of the yellow-colored fibroblast and all the other fibroblasts. Graphs show the distribution of the mutual distances between centroids at days 4, 7, 11, and 14 post-implantation (*n* = 5 regions in 3 mice).

Fibroblast is a non-immune key cell type involved in the FRB that contributes to the formation of the fibrotic capsule. Fibroblasts are characterized by complex shapes and different dimensions, and even for binary segmentation, neither simple thresholding nor more advanced rule-based techniques as morphological operators are able to perform their reliable segmentation. In such data domain, indeed, fibroblasts do not exhibit similar coherent intensities or simple geometry. Furthermore, no other automatic tool is available to detect and quantify these cells. In order to monitor fibroblast recruitment, including number and reciprocal spatial distribution, we trained the U-Net variant with RFP^+^ images and extracted these parameters. The automatic segmentation network accuracy was evaluated on the validation set upon training completion, reporting an average value of our custom metric of 0.982. Notably, the addition of batch normalization avoided early saturation of loss during training, regardless of the chosen objective, compared to the original U-Net architecture, as shown in Supplementary Figure S6. To validate the network performance, we manually quantified the number of fibroblasts in 7 images at low or high fibroblast density and correlated the outcome with predictions. The manual and predicted results showed a strong correlation (*R* = 0.702 and 0.950, respectively; Figures 4A,B). This segmentation approach was further validated by comparing the outcome derived from analysis with arivis Vision4D 3.5 and Icy. U-Net quantified the number of fibroblasts more accurately than both arivis Vision4D 3.5 and Icy, mostly at medium and high density (Supplementary Figure S7). While Icy and arivis Vision4D 3.5 could not distinguish between adjacent fibroblasts, U-Net, instead, was implemented with a loss function which emphasized cell separation (Ronneberger et al., 2015), as described in the Methods section. Geometrical parameters were then extracted for all the four different time points after scaffold implantation (*n* = 4 mice, 3 images/mouse). For each time point, fibroblasts were counted (Figure 4C) and their reciprocal distance calculated based on the centroid coordinates (Figure 4D), showing that the number of fibroblasts increased over time and their distance progressively decreased.

### 3.4 Automatic Quantification of Collagen Orientation

Activated fibroblasts secrete collagen to form a relatively thick fibrotic capsule around the implanted biomaterial, which is usually well oriented and has low vascular density (de Vos et al., 2006). Achieving a thinner capsule with randomly oriented collagen fibers by modifying material properties or through pharmacological inhibition would help minimize the FBR (de Vos et al., 2006). Thus, collagen orientation represents a key parameter to measure (Akilbekova and Bratlie, 2015). Fibrillar collagen is visualized through SHG detection (Figure 2C). This signal detects both polymeric proteins (e.g., fibrillar collagen) and PCL. (Dondossola et al., 2016). For these reasons, determining the orientation of biomaterial-induced collagen deposition with available tools (Rezakhaniha et al., 2012) was challenging, due to the confounding presence of scaffold fibers in the same input image. Thus, we adapted our U-Net variant to quantify collagen orientation and surrounding biological environment (Barad et al., 1997). Processing was performed as described in the Section 2.

**FIGURE 5:**
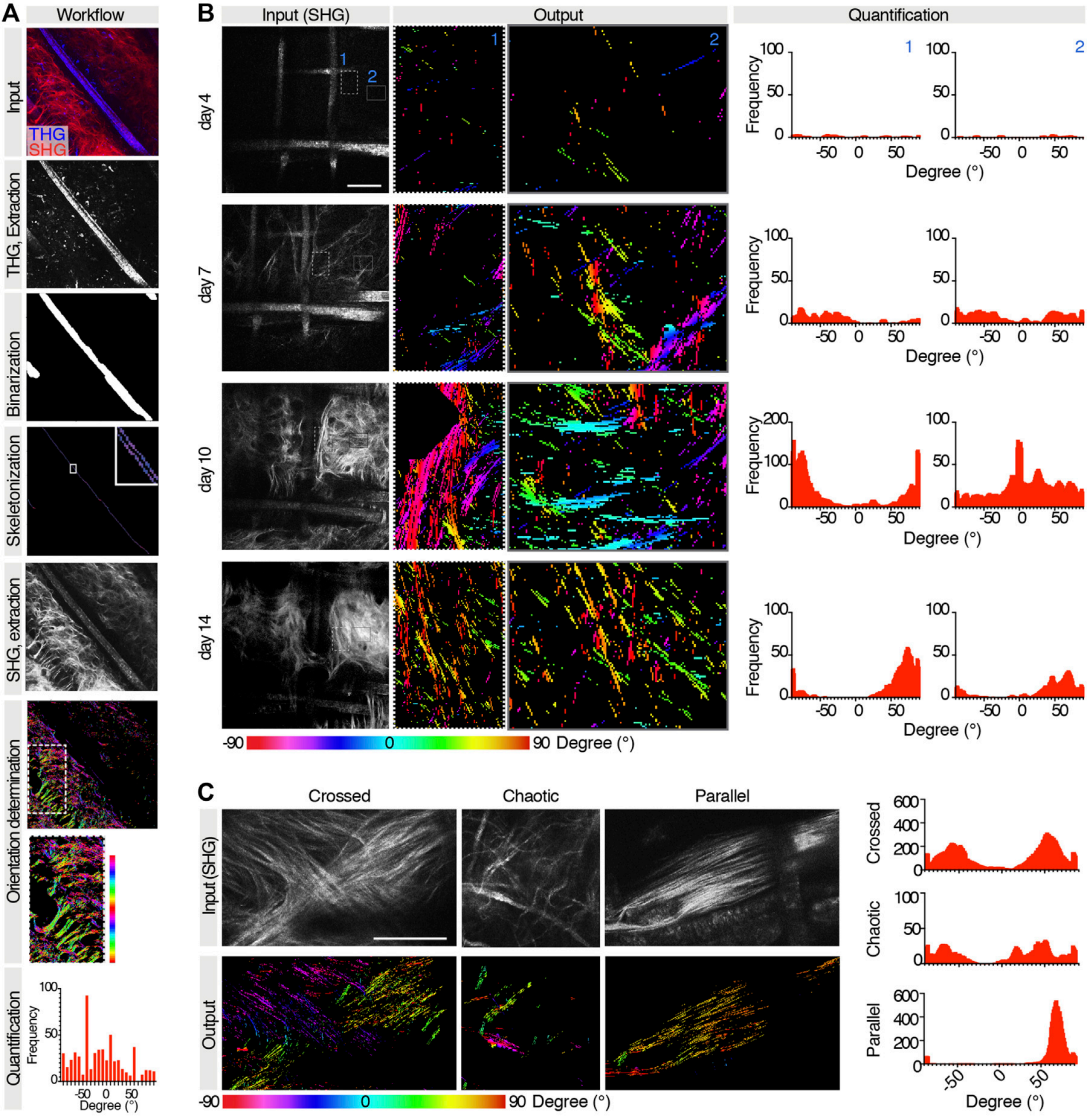
Extraction of collagen orientations relatively to the scaffold. **(A)** Workflow for extraction of collagen orientations relative to scaffold for a given case test. Top to bottom: raw input collagen and scaffold image (SHG and THG channels); THG channel extraction and skeletonization; SHG channel extraction and colormap of orientations of collagen boundaries in the whole image and in the subregion of interest where collagen directionalities are quantified; resulting histogram and frequency distribution relative to the predominant scaffold orientation. **(B)** Extraction of collagen orientations in the same region at days 4, 7, 11, and 14 post-implantation. For each time point, a raw input collagen image (gray), a color map of orientations of two subregions, and the relative histograms are shown. **(C)** Extraction of collagen orientations in regions with different patterns of collagen bundles. For each pattern, the raw input collagen image (gray), the output colormap, and the histogram of frequency are shown.

Information extraction was implemented relative to the main orientation of the scaffold framework, identified through THG signal. Briefly, the input image consisted of a binary SHG image displaying both scaffold and collagen, and a binary THG image displaying the scaffold only. THG images were skeletonized to identify the main orientation of the scaffold, then the THG signal was removed from SHG images, and the collagen orientation was determined. The resulting output was a spatial map and a histogram of pixel counts with tilting angle of attributed structures (Figure 5A). The scaffold segmentation network reported an IoU value of 0.796 over the validation set upon training completion, showing good detection accuracy. The tool was then tested to analyze the orientation of the collagen secreted in the same area overtime (Figure 5B). Interestingly, areas closer to PCL fibers showed an earlier normalization of collagen spatial distribution, with a prevalently parallel orientation, while areas at a greater distance from the scaffold assumed more random orientations. Then, as further validation purpose, our U-Net variant was applied to analyze three distinct patterns of orientation of the collagen bundles, defined as crossed, chaotic, or parallel (Figure 5C), which were correctly identified.

## 4 Conclusion

Three different applications were developed to endow scientists with automated software tools for performing multiparametric evaluation of the cellular and extracellular components of the FBR. All applications relied on deep learning, in particular on a U-Net variant, to accomplish semantic segmentation of structures of interest in respective input images with data-driven approaches, and parameters were extracted from predicted masks. So far, no applications were available to perform differential segmentation of immune subsets within the same image based on their topological distribution, for example, scaffold-associated cells *vs.* interstitial one, or to support segmentation of single fibroblasts and retrieval of parameters of interest.

The first application differentiated two cellular macro-populations (i.e., cells associated to the scaffold and interstitial cells) from acellular background within the same input image to quantify each subset, allowing for monitoring their recruitment over time and the consequences of therapeutic treatment. The second application quantified fibroblasts, including the number and reciprocal distribution. The third application allowed quantifying collagen orientation relatively to the segmented scaffold framework, thanks to implemented directional filtering based on customized kernels. For each application, images used to train and to validate the segmentation networks were preprocessed and manually labeled with expected results under biologists’ supervision. The limited datasets required by such models allowed for quick labeling, thanks to the extensive use of data augmentation, and fast training. The developed applications automated the workflow of scientists, yielding at the same time substantial time saving and reproducibility of results.

Concomitantly, the implemented applications proved our model versatility among different tasks. The employed network variant resembles the original U-Net architecture. The addition of normalization layers, and in particular of batch normalization (Ioffe and Szegedy, 2015), proved as determinant to avoid early loss saturation during training, regardless of the objective, as exemplified for fibroblast detection in Supplementary Figure S6. Results were not constrained by the employment of the same architecture and training hyperparameters across tasks. Conversely, validation metrics in all applications suggest compelling achievements considering the peculiar nature of the input images. IoU values are indeed highly dependent on the dataset and on the task difficulty, and values around 0.5–0.6 are considered a good measure of accuracy (Rezatofighi, 2019; Ulku and Akagunduz, 2019; Isensee et al., 2021). Application 1 (Section 3.2) metrics show similar values for both the scaffold-associated and the interstitial classes and report that the acellular background is rarely confused with cellular content. For application 2 (Section 3.3), the custom metric adopted to take into account both segmentation accuracy and enforcement of adjacent cell separation reflects the fact that the objective is also minimized on the validation set. Application 3 (Section 3.4) reports effective scaffold segmentation as well. Notably, segmentation results on the validation set strongly correlated with human labels for all tasks, suggesting reliable performances of this fully automated pipeline and boosting the hypothesis that the architecture can be versatile enough across different objectives. We did not need to tune any hyperparameter, that is, architecture and training setup, or the pre- or post-processing steps, obtaining considerable results across the three different and quite exhaustive common training objectives in semantic segmentation: the Dice loss, a pixel-weighted cross-entropy loss, and a new multiclass Dice extension. Interestingly, results confirm the trend of U-Net–like applications that very limited training sets guarantee reasonable performances, as usually required in the biomedical domain.

For collagen directionality quantification, several approaches have been developed which allow overall measurement of structures’ orientation (Rezakhaniha et al., 2012; Liu et al., 2020198; Liu et al., 2017). However, the presence of scaffolds and collagen fibers, together within the same channel, confounds the quantification and requires image preprocessing to either manually eliminate scaffold framework or cut out smaller areas of interest. Furthermore, several general filtering techniques were available with different configurable parameters each (e.g., a set of kernel kinds and kernel sizes for convolutional filtering), making it difficult to find an optimal setup for our images. As an improvement, by relying on the proposed U-Net variant for scaffold segmentation, our system automatically extracts information on collagen orientation only *via* dedicated filtering and reports it relatively to scaffold positioning. Besides monitoring the orientation of the collagen deposited within the scaffold fibers, this approach could be further used to determine the orientation of the external fibrotic capsule. Our convolutional filtering methodology allows defining linear edge detectors at the desired angular resolution and kernel size, to calibrate the gradient magnitude thresholds on the outputs and to apply variance filtering on such magnitudes, tuning the filtering to best suit collagen edges.

Blood vessel formation contributes to the FBR development (Dondossola et al., 2016; Gurevich et al., 2020). We did not address this aspect due to the great availability of tools that allow their segmentation and quantification (Yao et al., 2016; Fan et al., 2020; Mihelic et al., 2021).

While our U-Net resembles the original widely validated network, possible improvements include tuning architecture or training hyperparameters, such as the number of filters and layer blocks or mini-batch size and training epochs. To that end, cross-validation could be introduced without the need for an additional test set.

Our strategy is based on intravital acquisition of fluorescent reporter mice using a multiphoton microscope, an approach that is not easily available in research laboratories. However, we expect deep learning intrinsic adaptability to further allow applying our segmentation model to different data domains, with the possibility to extend analysis to 1) images acquired with different microscopes, for example, confocal or epifluorescence microscopes; 2) *ex vivo* 2D or 3D samples, including reconstructions of entire scaffolds or thinner slices; 3) antibody-based detection of specific markers, including immune cell subpopulations.

In this and a previous work (Dondossola et al., 2016), we did not identify significant differences based on mouse age or sex when comparing the FBR elicited by scaffolds implanted within the subcutaneous tissue. The progression of this process was consistent with other published work (Veiseh et al., 2015) which described implantation of biomaterials in different sites (intraperitoneal space) or in other species (non-human primates), although the extent and kinetics of cell recruitment over time might differ. As an advantage, the training of U-Net was performed with images that recapitulate the longitudinal development of the FBR, spanning both the initial stages (when cells are fewer) and later time points (when the cell density is higher), showing high efficiency by our U-Net variant in recognizing structures of interest during the validation process, as compared to manual analysis. For these reasons, we expect U-Net to be easily generalized; however, further validations could be performed after application of other biomaterials with compositions and geometries different from the regular grid of the PCL scaffolds in other implantation sites. In addition, the second and third applications could be extended to monitoring desmoplasia in other model systems, such as wound healing and other fibrotic processes, for example, pulmonary or tumor-induced fibrosis, including monitoring of fibroblast recruitment and collagen deposition, upgrading the recognition in the absence of a scaffold. We feel that our model application can be easily tailored to any future task of interest that requires different training objectives in similar scenarios. Overall, our promising automated software tool for the detection of cellular and extracellular structures and associated features will allow investigating FBR progression and aid to identify strategies that improve the performance of biomedical implants.

## Data Availability

The original contributions presented in the study are included in the article/Supplementary Material; further inquiries can be directed to the corresponding authors.
