# Supplementary material for: Deep Learning for Automated Analysis of Cellular and Extracellular Components of the Foreign Body Response in Multiphoton Microscopy Images
Source: Front Bioeng Biotechnol. 2022 Jan 25;9:797555. doi: 10.3389/fbioe.2021.797555 (PMC8822221; doi:10.3389/fbioe.2021.797555)
Supplement: Supplementary file 1 [file DataSheet1.docx]

**Deep learning for automated analysis of cellular and extracellular components of the foreign body response in multiphoton microscopy images**

**Supplementary figures**


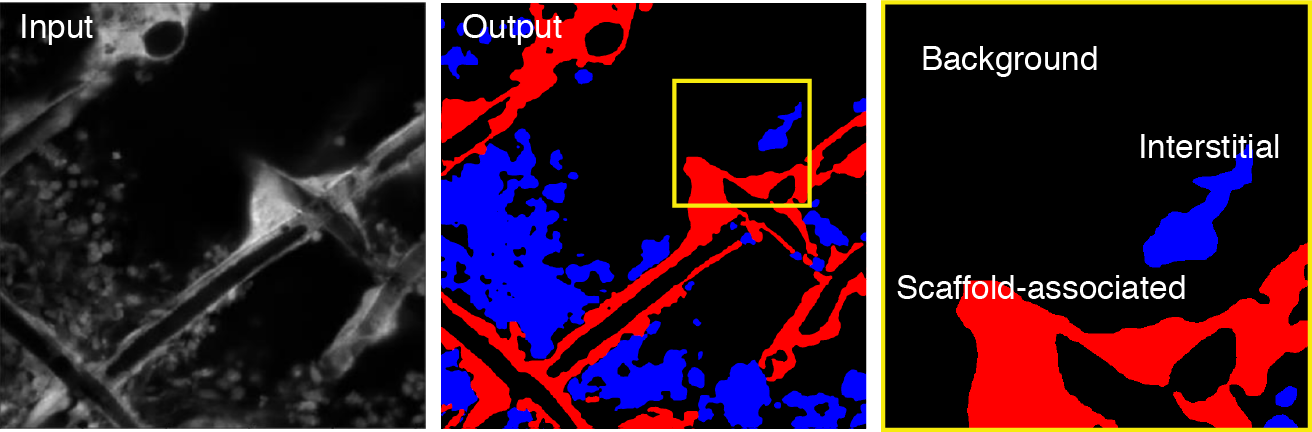


**Fig.S1. Elements recognized by U-Net multiclass segmentation.**


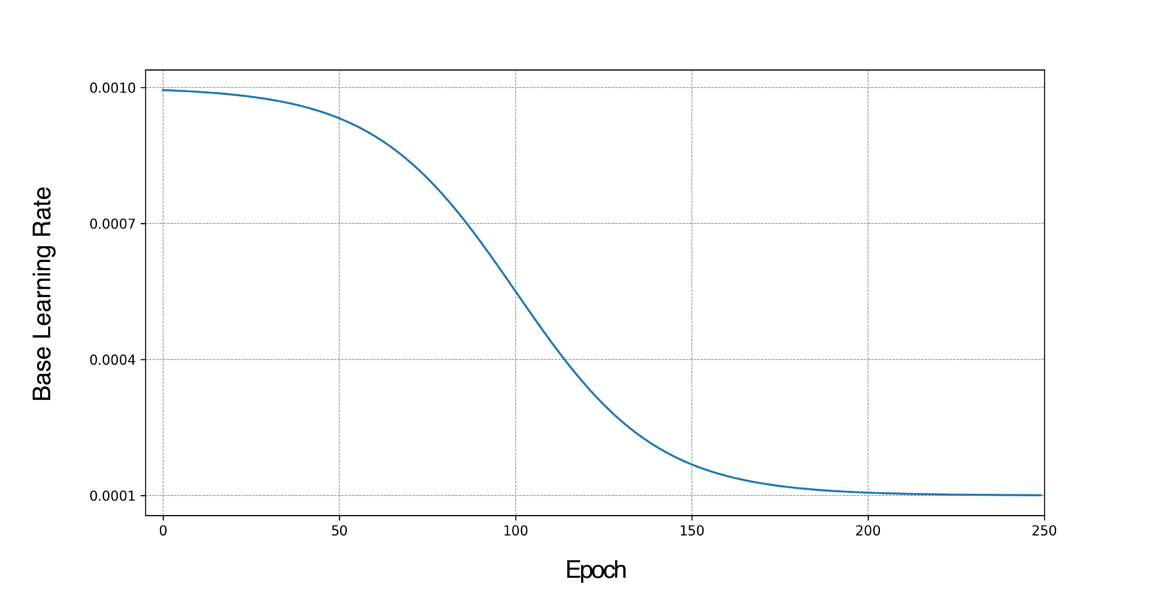


**Fig.S2. Base learning rate trend with training epochs.**


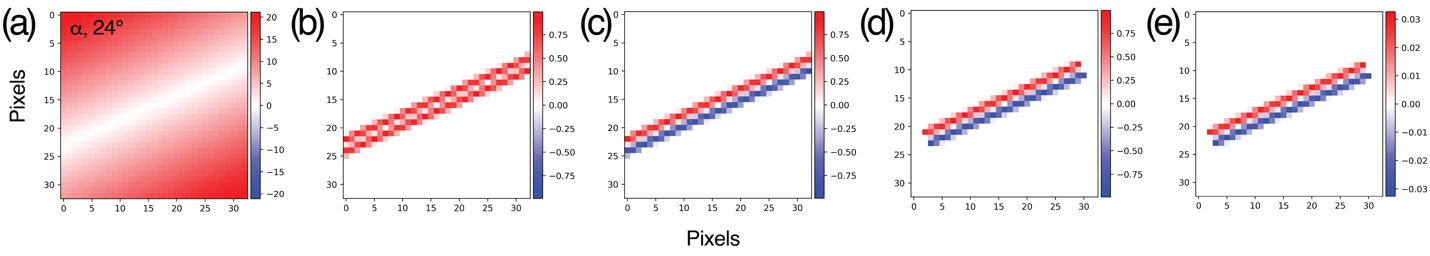


**Fig.S3. Kernel creation steps exemplified for an orientation of 24° (counterclockwise).** Coefficients cumulatively shown after: being assigned distance from central oriented line (a), ramp modulation and truncation of values (b), negative assignment of clockwise side values (c), truncation outside central radius (d) and normalization of both positive and negative values to unitary-sum modules (e)


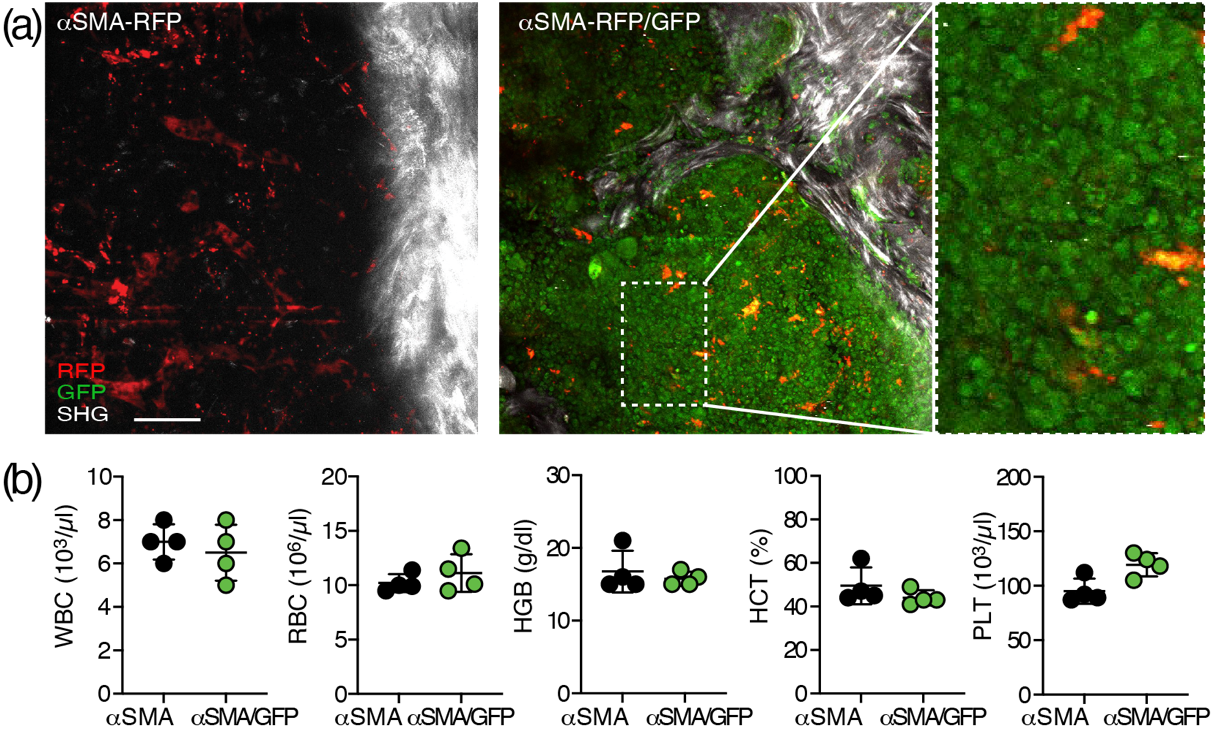


**Fig. S4. Confirmation of GFP bone marrow-derived cell engraftment.** (a) Immunofluorescence analysis of a bone from an αSMA and an αSMA-RFP/GFP mouse. Dashed box, inset. Scale bar, 100 μm. (b) Circulating white blood cells (WBC), red blood cells (RBC), hemoglobin (HGB) and platelets (PLT) as monitored 30 days post-bone marrow transplant. Mean + SD.


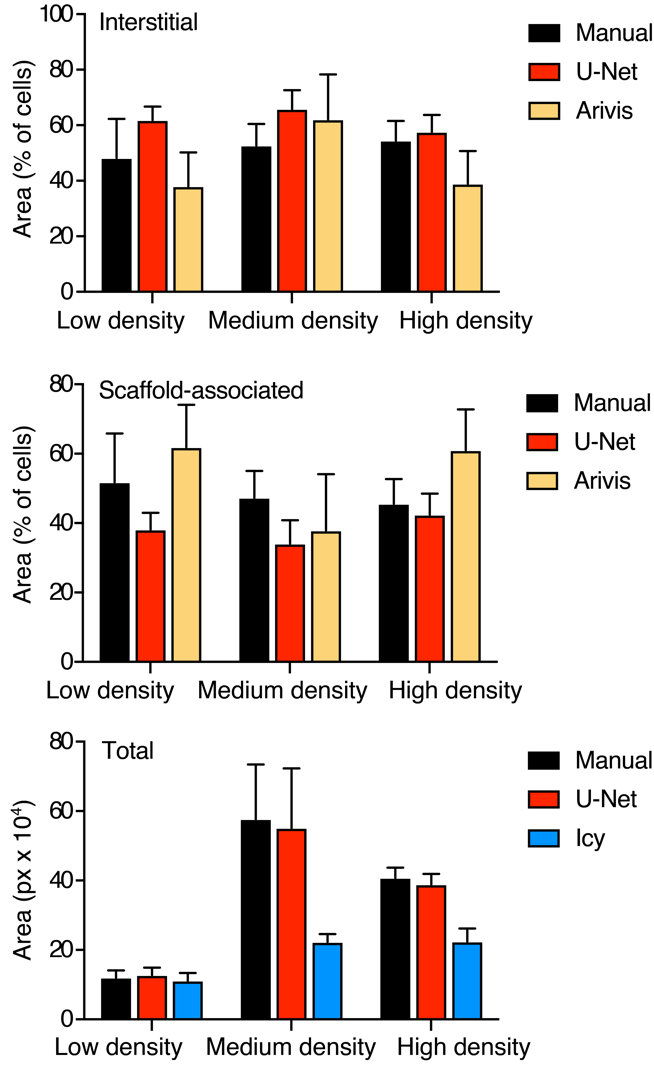


**Fig. S5. Comparison of immune-infiltrating GFP cell segmentation outcomes (area) by manual count, U-Net, Icy and Arivis Vision4D 3.5.**

**
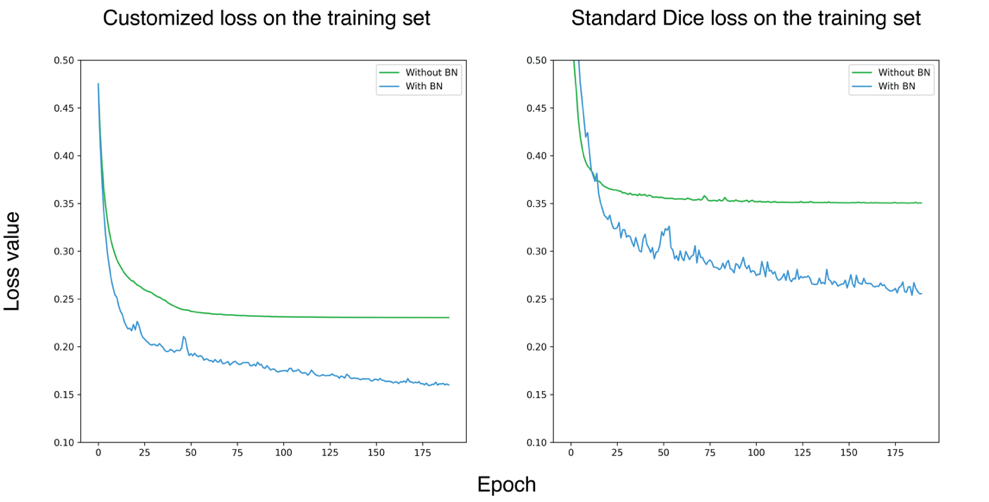
**

**Fig.S6. Batch Normalization Importance.** Our custom loss (left) and the standard Dice loss (right) on the training set with training epochs for the same network with (blue) and without (green) batch normalization layers: minimization is limited without batch normalization regardless of the objective.

**
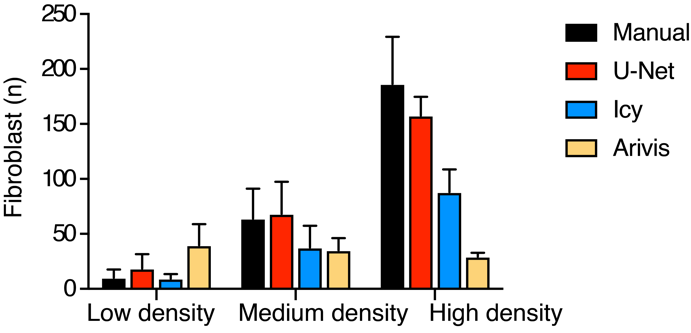
**

**Fig. S7. Comparison of fibroblast number identified by manual count, U-Net, Icy and Arivis Vision4D 3.5.**
